# A rare clinical image of fibromatosis colli on a 13-day-old baby

**DOI:** 10.11604/pamj.2025.52.11.47789

**Published:** 2025-09-10

**Authors:** Neha Krishnakumar Yadav, Sharath Hullumani

**Affiliations:** 1 Department of Community Physiotherapy, Ravi Nair Physiotherapy College, Datta Meghe Institute of Higher Education and Research, Sawangi (Meghe), Wardha, Maharashtra, India,; 2 Department of Paediatrics Physiotherapy, Ravi Nair Physiotherapy College, Datta Meghe Institute of Higher Education and Research, Sawangi (Meghe), Wardha, Maharashtra, India

**Keywords:** Myo-fibroblastic tumour, sternocleidomastoid muscle, benign

## Image in medicine

Fibromatosis colli (FC) is an uncommon pseudotumor of the sternocleidomastoid muscle, occurring with an incidence of 0.4%. It is often identified via ultrasonography between 2 and 4 weeks of age, with a higher prevalence in boys. It is categorized as a benign myofibroblastic tumour. A child about two weeks old with a unilateral sternocleidomastoid muscle hypertrophy is the typical presentation. The pathology seems to have a higher incidence in males. A 13-day-old boy was born via lower segment cesarean section (LSCS) breech presentation to a primi mother who was 31 weeks along in her pregnancy. After birth, the infant cried when stimulated and experienced mild respiratory distress and retractions; as a result, the baby was moved to the neonatal intensive care unit (NICU). Following ultrasound sonography (USG) results, fibromatosis colli is diagnosed.

**Figure 1:**
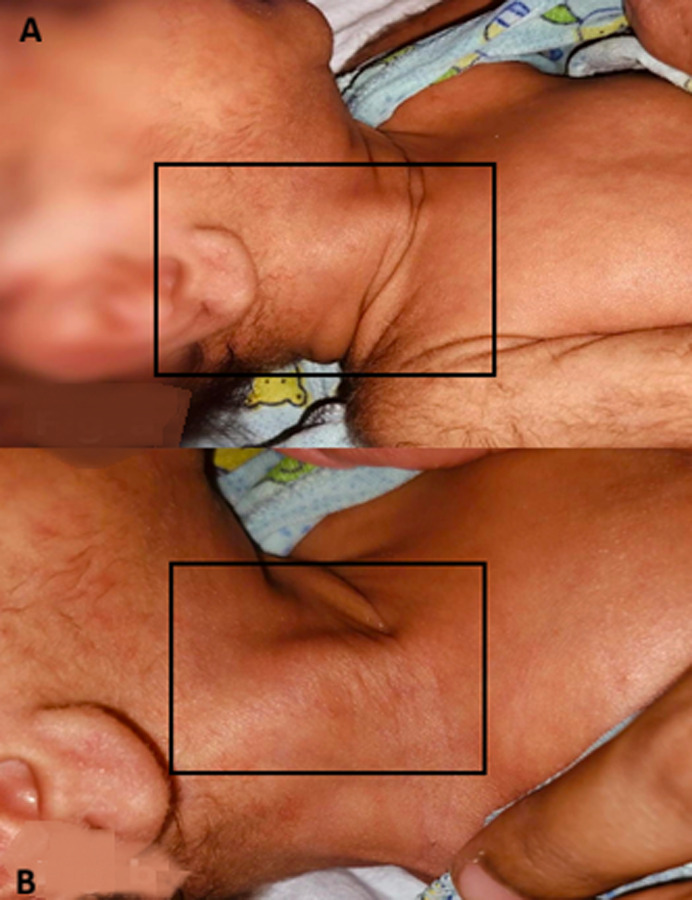
A,B) the palpable right neck abnormality demonstrates hypertrophy of the right sternocleidomastoid (SCM) muscle; the mass within the left SCM has heterogeneous echotexture, with fibromatosis colli; by comparison, the left (normal) SCM appears normal in texture; no aetiology of obvious collection noted; right thyroid gland appears normal, no aetiology of any focal lesion noted

